# Endemicity of *Coxiella burnetii* infection among people and their livestock in pastoral communities in northern Kenya

**DOI:** 10.1016/j.heliyon.2022.e11133

**Published:** 2022-10-21

**Authors:** Josphat Muema, Mutono Nyamai, Nick Wheelhouse, Joseph Njuguna, Christine Jost, Julius Oyugi, Zipporah Bukania, Harriet Oboge, Brian Ogoti, Anita Makori, Maria del Pilar Fernandez, Sylvia Omulo, S.M. Thumbi

**Affiliations:** a Institute of Tropical and Infectious Diseases, University of Nairobi, Nairobi, Kenya; b Washington State University Global Health Program – Kenya, Nairobi, Kenya; c Feed the Future Innovation Lab for Animal Health, Washington State University, USA; d Center for Epidemiological Modelling and Analysis, University of Nairobi, Nairobi, Kenya; e Edinburgh Napier University, Edinburgh, UK; f Food and Agriculture Organization of the United Nations, Nairobi, Kenya; g United States Agency for International Development's Bureau for Humanitarian Assistance (USAID/BHA), Washington, DC, USA; h Global Health Support Initiative III, Social Solutions International, Washington DC, USA; i Center for Public Health Research, Kenya Medical Research Institute, Nairobi, Kenya; j Paul G. Allen School for Global Health, Washington State University, Pullman, USA; k South African Center for Epidemiological Modelling Analysis, South Africa; l Institute of Immunology and Infection Research, University of Edinburgh, UK

**Keywords:** Endemicity, *Coxiella burnetii*, Q-fever, People, Livestock, Pastoral, Kenya

## Abstract

**Background:**

*Coxiella burnetti* can be transmitted to humans primarily through inhaling contaminated droplets released from infected animals or consumption of contaminated dairy products. Despite its zoonotic nature and the close association pastoralist communities have with their livestock, studies reporting simultaneous assessment of *C. burnetti* exposure and risk-factors among people and their livestock are scarce.

**Objective:**

This study therefore estimated the seroprevalence of Q-fever and associated risk factors of exposure in people and their livestock.

**Materials and methods:**

We conducted a cross-sectional study in pastoralist communities in Marsabit County in northern Kenya. A total of 1,074 women and 225 children were enrolled and provided blood samples for Q-fever testing. Additionally, 1,876 goats, 322 sheep and 189 camels from the same households were sampled. A structured questionnaire was administered to collect individual- and household/herd-level data. Indirect IgG ELISA kits were used to test the samples.

**Results:**

Household-level seropositivity was 13.2% [95% CI: 11.2–15.3]; differences in seropositivity levels among women and children were statistically insignificant (*p* = 0.8531). Lactating women had higher odds of exposure, odds ratio (OR) = 2.4 [1.3–5.3], while the odds of exposure among children increased with age OR = 1.1 [1.0–1.1]. Herd-level seroprevalence was 83.7% [81.7–85.6]. Seropositivity among goats was 74.7% [72.7–76.7], while that among sheep and camels was 56.8% [51.2–62.3] and 38.6% [31.6–45.9], respectively. Goats and sheep had a higher risk of exposure OR = 5.4 [3.7–7.3] and 2.6 [1.8–3.4], respectively relative to camels. There was no statistically significant association between Q-fever seropositivity and nutrition status in women, *p* = 0.900 and children, *p* = 1.000. We found no significant association between exposure in people and their livestock at household level *(p =* 0.724) despite high animal exposure levels, suggesting that Q-fever exposure in humans may be occurring at a scale larger than households.

**Conclusion:**

The one health approach used in this study revealed that Q-fever is endemic in this setting. Longitudinal studies of Q-fever burden and risk factors simultaneously assessed in human and animal populations as well as the socioeconomic impacts of the disease and further explore the role of environmental factors in Q-fever epidemiology are required. Such evidence may form the basis for designing Q-fever prevention and control strategies.

## 1 Introduction

Q-fever, caused by the bacterium *Coxiella burnetii*, is an infectious zoonotic disease with worldwide occurrence except in New Zealand and the Antarctica [1]. Domestic ruminants including cattle, goats, and sheep are the main sources for human infection [2]. Camel-associated human infections have recently been reported [3].

Q-fever transmission occurs primarily through the inhalation of aerosols from contaminated birth materials of infected animals [4] and from the contaminated environments [5]. Other transmission route include through wind dispersal [6, 7], consumption of unpasteurized milk [8, 9] or bites from infected ticks [10]. In humans, Q-fever manifests as flu-like illness or atypical pneumonia, which can progress to acute respiratory distress syndrome [11]. In animals, it is mostly asymptomatic although reproductive disorders have been reported [12, 13]. Human *C. burnetii* exposure risk factors include occupational exposure [14], engagement in small ruminant farming [15], lack of formal education [16], being of a male gender, involvement in camel breeding [3], and being of the young age category [3, 17]. Risk factors for seropositivity to Q-fever in animals include older age, female gender, and extensive livestock production system/nomadic pastoralism [18, 19, 20].

Despite the significant health and economic impacts associated with this disease [21], Q-fever remains a neglected zoonotic disease [22] which requires multidisciplinary One Health approach to address [23, 24]. A systematic review by Vanderburg et al., identified evidence gaps on Q-fever burden, geographical spread and the risk factors for *C. burnetii* infection in Africa [25]. The review identified seroprevalence estimates ranging from 4% to 55% in cattle, 11%–33% in sheep, 13%–24% in goats and 1%–32% in humans in various studies from Africa [25]. However, linked human-animal population studies were scarce with only two studies reported, one in Egypt [26] and another in Chad [3]. This dearth in studies accessing the prevalence and risk factors for *C. burnetii* infection in human and animal populations concurrently limits our understanding of the epidemiology of Q-fever in the African region.

In Kenya, Q-fever is ranked among the top ten priority zoonotic diseases [27]. Nevertheless, a recent systematic review [28] indicates that high quality data on the disease burden and its transmission dynamics are scarce. Few cross-sectional surveys suggest that Q-fever is endemic in parts of Kenya. For example, Knobel et al. [29], reported a seroprevalence of 28% in cattle, 32% in goats, 18% in sheep and 31% in humans, implicating ticks as potential vectors for *C. burnetii* transmission in rural western Kenya [29]. Another study in western Kenya, reported a seroprevalence of 10.5% in cattle and 2.5% humans, highlighting the role of environmental factors in *C. burnetii* exposure to cattle [13]. Other studies have reported heterogeneity in Q-fever seroprevalence ranging from 0% to 4% in cattle, 13–20% in sheep, 31–40% in goats and 5–46% in camels, with seropositivity in camels increasing with age [30, 31].

Pastoral communities, primarily located in northern Kenya, are at an increased risk of exposure to *C. burnetii* due to increased contact with livestock and high livestock densities [11]. Nevertheless, the prevalence of Q-fever and the transmission dynamics of the disease within this population has not been characterized. We estimated the seroprevalence of *C. burnetii* simultaneously in both human and domestic ruminant populations in a pastoral community, determine the risk factors for. and associations between *C. burnetii* exposure in humans and domestic ruminants. For the human population, the study focused on children below five years and women of reproductive age because part of the objective was to identify the association of Q-fever exposure status and the nutritional status of these two sub-populations that are most nutritionally vulnerable. The study sampled only female animals as the target was lactating animals providing milk to the households. The study was conducted during a dry season, and majority of the households had small ruminants and camels for milking purposes as these animals are considered more drought resilient compared to cattle [32]. In this setting only a small proportion of the population keep cattle which had migrated to dry season grazing areas satellite camps (‘fora’) in search of water and pasture, hence the field team sampled only goats, sheep, and camels. Goats were the majority of the sampled animals (78%) as they were the animals most households relied on for milk provision to the households during the dry season. The simultaneous assessement of Q-fever burden and associated factors in human-animal populations allowed us to examine individual and household/herd-level associations between animal and human exposure and to explore shared risk factors.

## 2 Materials and methods

### 2.1 Study area

**Figure 1:**
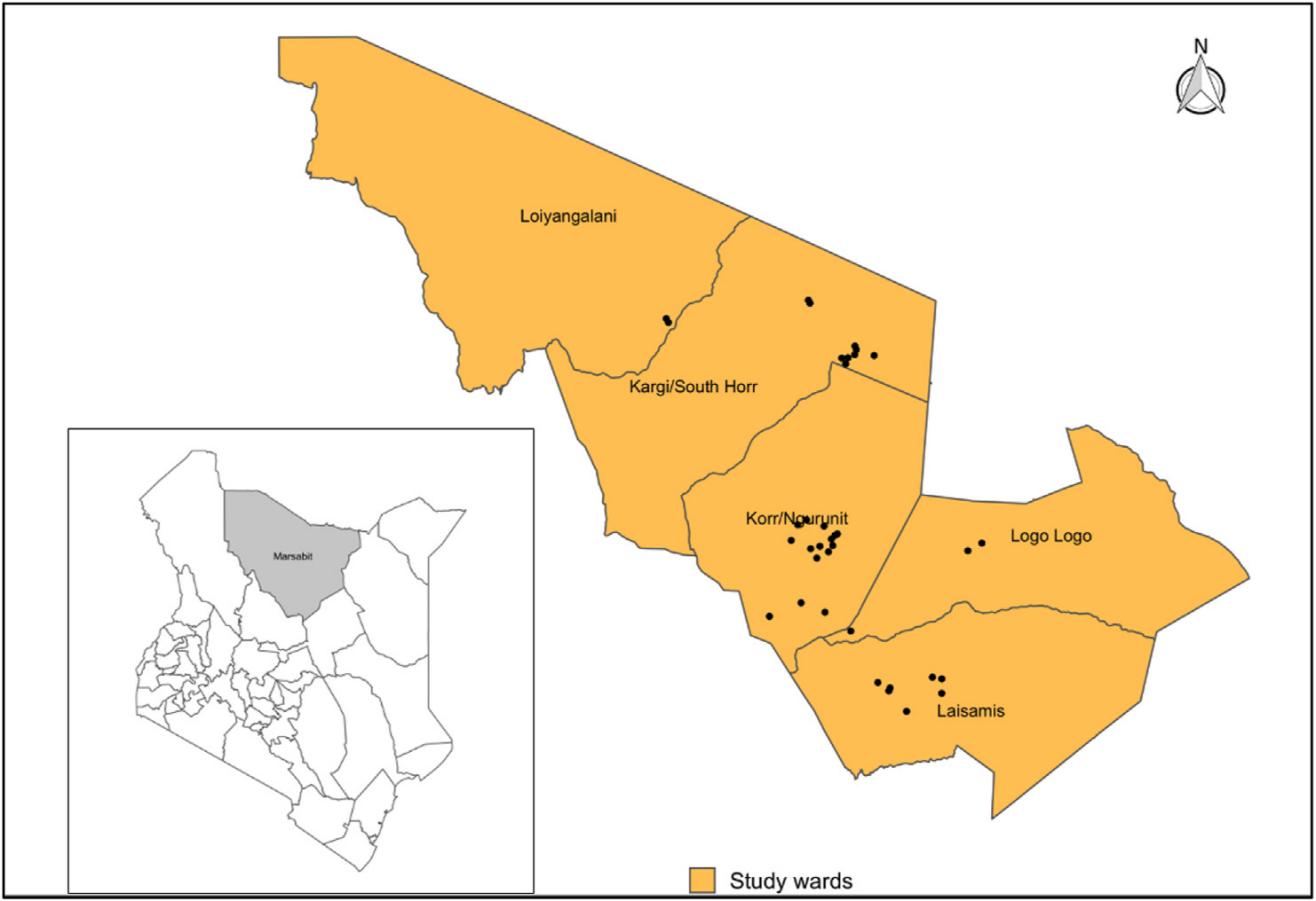
Map showing the position of Marsabit County within Kenya (left), Laisamis sub-county and wards included in the study indicating all sampled villages (Black dots). Shapefile source: GADM.

This study was conducted in Laisamis subcounty of Marsabit County between September and November 2019. Marsabit county, which is located in northern Kenya, is predominantly a nomadic pastoralist region (Figure 1). The study was specifically conducted in parts of Logologo, Laisamis, Kargi, Korr and Loiyangalani wards with similar climatic conditions.

### 2.2 Sample size and sampling

The sampling piggy-backed on a larger research project, Livestock for health (L4H) project, which is a cluster randomized controlled trial investigating the effect of livestock supplementary feeding intervention during dry periods and nutrition counselling on maternal and child nutrition in northern Kenya. The study population was composed of women of child bearing age, children <5 years and livestock providing milk to the households. This population was chosen because women of reproductive age especially pregnant and lactating women and children <5 years of age are the most nutritionally vulnerable group and are a good indicator of a household nutritional status. We investigated the burden of Q-fever in the same population since high prevalences of Q-fever have been reported in similar pastoral production systems in kenya [20, 33, 34] and due to its chronic debilitating sequelae, we wanted to determine if its associated with the high rates of malnutrition reported in women and children in this setting.

In brief, A multi-stage cluster sampling was conducted to select study participants. All the five wards within the Laisamis subcounty namely Logologo, Laisamis, Kargi, Korr and Loiyangalani were included and a list of all sublocations within these wards generated. Twelve sublocations were randomly selected for the study and a list of all villages within each of the selected sublocations generated and used as a sampling frame to randomly select three villages per sublocation. In each village, households with a lactating animal, child less than five years and woman of reproductive age were eligible for inclusion in the study.

The primary sampling unit was the household while the secondary sampling units were children <5 years, Women of child bearing age and the individual animals within the household herd. A household herd was defined as an aggregate of livestock (cattle, goats, sheep, and camels (dromentary one humped camels)) under the same management system. We assumed that household herds are exposed to common risk factors for disease and that disease distribution within a household herd was homogenous. We applied an expected herd prevalence of 50%, a desired absolute precision of 5%, and test sensitivities and specificities of 95% and 99%, respectively, to obtain a minimum sample size of 960 households. We chose the 50% prevalence because it provides the largest sample size for given values of absolute error.

In each household herd, a maximum of three lactating animals per species were randomly selected systematically using a sampling interval number obtained by dividing the total number of lactating animals per species by number of animals to be sampled within the herd. The first animal was randomly selected followed by every *n*th animal until the sample size was attained. In each household herd, all lactating animals per species were grouped together and all the lactating animals within the household herd were numbered using animal marker pens and random numbers assigned by dividing the total number of lactating animals per species by three (3) to create the interval of selection. Animals bearing the random number were selected for blood sample collection. For the human participants children aged <5 years and women of reproductive age within the households who had consented to be part of the larger livestock for health project in which this study is nested were enrolled for blood sample collection. The distribution of samples per ward and per species is provided (Table1).

**Table 1:** Distribution of samples per ward per species.

	Ward	Number of samples per village per species			
		Goats	Sheep	Camels	Human
	Korr/Ngurunit	558	254	102	492
	Laisamis	449	25	28	332
	Logologo	333	12	14	242
	Loiyangalani	174	9	34	16
	Kargi/SouthHorr	362	22	11	217
	Total	1876	322	189	1299

### 2.3 Survey data collection

Data on household-level attributes including demographic characteristics, herd health and management, and grazing distance were abstracted from a the livestock for health project baseline survey conducted on this population prior to this study and in which this study is nested.

The human level factors considered in the study included age, sex, and physiological status and were collected through a human sample collection and tracking questionnaire (annex 1) administered to the women of reproductive age from which blood samples were collected. Human nutrition status was abstracted from a the livestock for health project baseline survey conducted on this population and in which this study is nested.

Animal level attributes including species, age, sex, and history of reproductive disorders were collected through an animal-level sample collection and tracking questionnaire (annex 2) administered to the household head or the person taking care of the animals.

### 2.4 Sample collection

Venous blood was collected from humans and animal by trained nurses and animal health technicians, respectively. Human samples were collected in plain 5 ml vacutainers while animal samples in 10 ml vacutainers. For the human samples, 2.5 ml of venous blood was collected from children and 4 ml from women while for the animal samples, 8 ml of blood was collected from goats, sheep and camels. All samples were barcoded and allowed to stand for 15 min to allow clot separation, then transported in cooler boxes to a field laboratory within 6 h of collection.

### 2.5 Laboratory procedures

To harvest serum, samples were centrifuged at 3000×*g* for 10 min. Serum was collected in 2 ml cryovials and stored at −20 °C until transported to the University of Nairobi Institute of Tropical and Infectious Diseases (UNITID) laboratory in Nairobi for testing.

Samples were tested for *Coxiella burnetti* antibiodies using indirect ELISA test kits. Human sera were tested using the SERION ELISA Classic *Coxiella burnetii* phase 2 IgG (SERION Diagnostics, Würzburg, Germany) kit, which has a sensitivity of 92.5% and specificity of >99%. Animal sera were tested using the PRIOCHECK™ Ruminant Q Fever IgG (ThemoFisher Scientific, UK) ELISA kit which has a sensitivity of 87% and specificity of 99.1%. Testing was done following manufacturer's instructions. Human sample ODs were read at 405 nm and a reference wavelength of 630nm on a HumaReader HS microtiter plate reader, and results interpreted based on manufacturer's recommendations. Animal sample ODs were read at 450 nm and interpreted as negative or positive based on percent positivity (PP) cutoff values of <40 or >40, respectively.

### 2.6 Data analysis

Logistic regression models were used to identify individual- and household/herd level factors associated with *C. burnetii* antibody seropositivity. A univariable model was used to explore the relationship between Q-fever seropositivity and independent predictor variables. The independent predictor variables assessed for human models included age, sex, physiological status, occupation, education level, geographical location (ward) and nutritional status. For the animal models the independent variables assessed included species, geographical location (ward), reproductive disorders, household head occupation, household head education level and grazing distance, All predictor variables were added to a multivariable model and a variable selection for the final model carried out using the stepwise Akaike Information Criterion algorithm. Odds ratios and corresponding 95% confidence intervals were calculated to identify the strength of identified associations. The fitted models were evaluated by including household/herd as a random effect to adjust for possible clustering of *C. burnetii* seropositivity within households/herds. Model diagnostics included calculating scaled residuals, mapping residuals, and testing for dispersion and spatial autocorrelation of residuals. Model building assumed family binomial with logit link functions. All analyses were performed using R version 3.6.2 [35].

### 2.7 Ethics statement

Ethical approval was obtained from the Kenya Medical Research Institute Scientific and Ethics Review Unit (KEMRI/SERU/CGHR/02-09/3755) and the Kenyatta National Hospital/University of Nairobi Ethics and Research Committee (KNH-ERC/A/69-P850/10/2019) for collection of both human and animal samples. Written informed consent was obtained from the study participants prior to enrollment and data collection. For minors (children <5 years of age) written parental/legal guardian permission was obtained prior to sample collection. All animal owners provided a signed informed consent before specimen collection. The animal restraint and sampling were designed to be less invasive for both animal and personal safety and were conducted by animal techniciations and veterinary surgeons according to the World Organization for Animal Health (OIE) guidelines for use of animals in research and education [36].

## 3 Results

### 3.1 Socio-demographic characteristics of human and animal study population

A total of 1,734 households who had been enrolled in the larger livestock for health project study trial were approached for enrollment, out of which 1,095 (63%) households agreed to participate in the Q-fever study. From these 1,095 households, a total of 1,299 participants were enrolled and provided samples, 1,074 (83%) of whom were women and 225 (17%) children. The mean age of enrolled women was 28.6 years (range: 17–46), while that of children was 23.4 months (range: 5–42). Among women, 905 (84.3%) were lactating while 169 (15.7%) were not lactating. All households owned at least one livestock species (goats, sheep, camels and cattle) with ownership of goats at 96%, sheep (92%), camels (68%), cattle (43%), donkeys (60%) and chicken (13%). On average, the households had three camels, seven goats, six sheep and three cattle. In total, 2,387 animals were sampled including 1,876 (78%) goats, 322 (14%) sheep and 189 (8%) camels. No cattle were sampled as the few cattle kept by the communities were in dry season grazing areas.

### 3.2 Household level seroprevalence

A total of 144 of 1,095 households had at least one seropositive individual, resulting in household level seroprevalence of 13.2% [95% CI: 11.2–15.3].

### 3.3 Seroprevalence estimates of Q-fever in women and children

The *C. burnetii* antibody seropositivity among women was 121/1,074, resulting to a seropositivity of 11.3% [9.4–13.3] while that among children was 30/225, giving a seropositivity of 13.3% [9.2–18.5]. Seroprevalence varied with socio-demographic characteristics (Table 2). Age was included as continuous variable to determine its effect on the study outcome in both women and children. Age was not significantly associated with Q-fever seropositivity (*p* = 0.857). Age was only significantly associated with Q-fever seropositivity in children OR = 1.1 (1.0–1.1), *p* = 0.049.

**Table 2:** Q-fever seroprevalence in women and children by sociodemographic characteristics and results of univariable analysis.

	Women (N = 1,074)		Univariable analysis	Children (N = 225)		Univariable analysis
Variable	n/N (%)	95% CI	p-Value	n/N (%)	95% CI	p-Value
*Occupation*						
Livestock herding	91/728 (12.5)	10–15	0.86		–	–
Employment/business	29/318 (9.1)	6–12			–	–
*Physiological status*						
Lactating	112/905 (12.4)	10–15	0.026		–	–
Non-lactating	9/169 (5.3)	3–10			–	–
*Education level*∗						
Formal education	8/86 (9.3)	4–18	0.548	3/17 (17.6)	4–43	0.586
No formal education	113/988 (11.4)	10–13		27/208 (13.0)	9–18	
*Location (ward)*						
Kargi/SouthHorr	27/209 (12.9)	9–18	0.378	0/8 (0.0)	–	0.408
Korr/Ngurnit	52/426 (12.2)	9–16		10/66 (15.2)	8–26	
Laisamis	28/260 (10.8)	7–15		7/72 (9.7)	4–19	
Logologo	14/163 (8.6)	5–14		13/79 (16.5)	9–27	
Loiyangalani	0/16 (1.5)					
*Nutritional Status*						
Malnourished	14/128 (12.9)	6–18	0.900	6/45 (13.3)	5–27	1.000
Normal	107/946 (11.3)	9–14		24/180 (13.3)	8–19	

∗ For children, this refers to mother’s education level.

### 3.4 Herd level level seroprevalence

Of the 1,443 herds sampled, 1,208 herds had at least one seropositive animal, yielding a herd seroprevalence of 83.7% [81.7–85.6].

### 3.5 Individual animal level seroprevalence estimates

The overall seroprevalence in sampled animals was 69.5% [67.6–71.3], with species seroprevalence of 74.7% [72.7–76.7] among goats, 56.8% [51.2–62.3] among sheep and 38.6% [31.6–45.9] among camels. Seroprevalence in animals varied by sociodemographic characteristics (Table 3). Age was included as continuous variable and was not associated with Q-fever seropositivity in animals (p = 0.9118).

**Table 3:** Q-fever seroprevalence in animals by socio-demographic characteristics and univariable analysis results.

Overall seroprevalence 1658/2387 (69.5%)			Univariable analysis
Variable	Seroprevalence n/N (%)	95% CI	p-Value
*Geographical location (Ward)*			
Kargi/Southhorr	280/395 (70.9%)	66.1–75.3	<0.001
Korr/Ngurunit	586/931 (62.9%)	59.8–66.1	
Laisamis	396/520 (76.2%)	72.3–79.8	
Logologo	256/350 (73.1%)	68.2–77.7	
Loiyangalani	140/191 (73.3%)	66.4–79.4	
*Species*			
Goats	1402/1876 (74..7%)	72.7–76.7	<0.001
Sheep	183/322 (56.8%)	51.2–62.3	
Camels	73/189 (38.6%)	31.7–45.9	
*Reproductive disorders*			
No	1145/1641 (69.8%)	67.5–71.9	0.620
Yes	513/746 (68.8%)	65.3–72.1	
*Household head Occupation*			
Livestock herding	1216/1789 (68.0%)	65.8–70.1	0.115
Employment/business	442/598 (73.9%)	70.2–77.4	
*Household head Education*			
No formal education	1299/1882 (69.0%)	66.9–71.1	0.549
Formal education	119/167 (71.3%)	63.8–77.9	
*Grazing distance*			
<5 km	459/662 (69.3%)	65.7–72.8	0.182
5–10km	536/746 (71.8%)	68.5–75.1	
>10km	663/979 (67.7%)	64.7–70.7	

### 3.6 Risk factors associated with Q-fever seropositivity in women and children

Multivariable models showed significant associations between *C. burnetii* seropositivity and the physiological status of a woman (lactation), with the likelihood of exposure being 2.4 [1.3–5.3] folds higher in lactating women than in non-lactating women (*p* = 0.013). Among children, age was significantly associated with seropositivity, with the odds of seropsositivity increasing by 1.1 [1.0–1.1] for every unit increase in age (Table 4).

**Table 4:** Risk of being *C. burnetii* antibody seropositive in women and children.

Women	Multivariate analysis		Children	Multivariate analysis	
Variable	OR (CI)	P value	variable	OR (CI)	P value
*Household head occupation*			*Age*	1.1 (1.002–1.1)	0.049
Livestock herding	1.4 (0.92–2.23)	0.126	–	–	
Employment/business	Ref				
*Physiological status*			*Sex*		
Lactating	2.4 (1.28–5.28)	0.013	Male	0.4 (0.16–1.1)	0.078
Non-lactating	Ref		Female	Ref	

### 3.7 Risk factors associated with livestock seropositivity for Q-fever

The likelihood of seropositivity to *C. burnetti* was 5 [3.8–7.8] and 3 [1.8–4.0] folds higher in goats and sheep, respectively, relative to seropositivity in camels. Statistically significant differences in *C. burnetii* antibody seroprevalence were observed among animals from different wards in the study area, with animals from Laisamis and Loiyangalani wards being respectively 1.4 fold more likely and 1.7 fold more likely to be seropositive compared to Kargi/South Horr (Table 5). Animals from households where the household head main occupation was livestock herding had less odds of being seropositive OR = 0.56 (CI 0.4–0.8), *p* = 0.003 compared to those engaged in employment/business.

**Table 5:** Risk of being *C. burnetii* antibody seropositive in animals.

	Multivariate analysis	
Variable	OR (95% CI)	P value
Animal-level factors		
Geographical location (Ward)		
Korr/Ngurunit	1.0 (0.7–1.4)	0.956
Laisamis	1.4 (1.0–1.9)	0.047
Logologo	1.1 (0.8–1.6)	0.590
Loiyangalani	1.7 (1.1–2.8)	0.017
Kargi/SouthHorr	Reference	
Species		
Goats	5.5 (3.9–7.8)	<0.001
Sheep	2.7 (1.8–4.0)	<0.001
Camels	Reference	
Herd- level factors		
Household head Occupation		
Livestock herding	0.6 (0.4–0.8)	0.003
Employment/business	Reference	
Household head formal education		
Yes	0.7 (0.5–1.1)	0.099
No	Reference	

### 3.8 Association between Q-fever seropositivity and nutrition status in women and children

When nutritional status was added in both the women and children individual level models, there was no statistically significant association between Q-fever seropositivity and nutrition status in women, *p* = 0.900 and children, *p* = 1.000.

### 3.9 Association between Q fever seropositivity in people and their livestock

We did not find statistically significant association between Q-fever seropositivity in people and the livestock they kept when the association was tested at household level *(p =* 0.724).

## 4 Discussion

Our study reports a high prevalence of *C. burnetii* in domestic ruminants with more than two thirds of goats and sheep, and more than a third of camels previously exposed. We also report a high prevalence of *C. burnetti* in people with exposure levels in children (13.3%) similar to those observed in adults (11.3%) suggesting a high infection pressure in the study region of northern Kenya. By studying both people and their livestock, we explore the associations between exposure status in animals and in people and do not find clear results suggesting a direct association at household level. Further, we explored factors associated with increased risk of *C. burnetii* exposure in both human and domestic ruminants’ population and examined the implications of our findings to disease burden, spread and control strategies.

There were no statistically significant differences in *Coxiella burnetii* exposure levels among children <5 years and women of reproductive age. This could possibly be due to high *Coxiella burnetii* infection levels in this setting making the probability of exposure between children and adults almost similar as children are exposed to *Coxiella burnetii* early in life [16]. The exposure to *C. burnetii* in children has been reported elsewhere [16]. Our results differ with previous studies which showed greater risk in older people and attributed it to cumulative risk of exposure in older people compared to children [34]. However, children naïve immune response could be a predisposing factor. Furthermore, our results suggest that Q-fever is not just an occupational hazard among adults but also affects children. Lactating women had higher odds of exposure compared to non-lactating women. *C. burnetii* has been isolated from breast milk previously [37, 38, 39]. However, the pathogenic role of *C. burnetiid* in lactating women is still uncertain. Further research on the role of physiological status including pregnancy and lactation in Q-fever transimission dynamics is plausible.

Very high seroprevalence was recorded in animals compared to humans where for every 100 animals sampled, at least 69 of them had been previously exposed. Goats had the highest seroprevalence with three-quarters of the sampled goats having been exposed to Q-fever compared to 57% of sheep and 37% in camels. This could be associated probably with environmental exposure with goats being browzers and closer to the ground compared to camels hence higher risk of exposure through conterminated environment. Future studies in this setting should consinder environmental sampling. At herd level, for every ten herds sampled at least eight had an animal positive for Q-fever antibodies. The results indicate the disease is endemic in animal and human populations in this setting. A study by Larson and others found *C. burnetii* seroprevalence estimates of 20% in cattle, 18% in goats and 13% in sheep in Laikipia county [40]. Another study conducted in two arid and semi-arid (ASAL) counties of Isiolo and Samburu found a *C. burnetii* seroprevalence of 21% in camels [41]. A recent systematic review conducted in Kenya recorded evidence of *C. burnetii* infections ranging from 7%–20% in sheep, 20%–46% in goats and 20%–46% in camels in Kenya [28]. Our study recorded higher seroprevalence estimates in animals compared to previously conducted studies in the country. However, our study focused on lactating animals proving milk to the households which were all female and older animals. Previous studies have shown higher seroprevalence estimates in female animals as well as older animals [20, 42, 43]. All our sampled animals were female and female animals are more likely to be in a closer proximity to birth products the primary route of infection as well as being older compared to general population.

Significant differences in apparent exposure levels to *C. burnetii* were observed among the animals included in this study. Our multivariable analysis revealed that seroprevalence varies by species, geographical location (ward) and the main occupation of the household head for the combined goats, sheep and camels data. Seropositivity across the three sampled species was heterogenous with goats being 5.4 folds and sheep 2.6 folds likely to be seropositive compared to camels. The results indicate goats are an important species in the transmission dynamics of *C. burnetii* in this region. Other studies have found similar trends in Kenya and by extension the African continent where high exposure levels have been found in goats compared to sheep [20, 44, 45, 46]. However other studies have also recorded higher seroprevalence estimates in sheep compared to goats [47, 48, 49] hence further research is required to understand the inherent differences in *C. burnetii* transmission dynamics among small ruminants.

Several studies have documented age as a determinant of *C. burnetii* exposure in animals, where increasing age is associated with increased odds of being *C. burnetii* antibody seropositive [31, 50, 51, 52, 53]. However, in our study, age was not statistically associated with *C. burnetii* antibody seropositivity. This could be partly due to the choice of our study animal population which were mainly lactating animals whose age structure may not be very different hence the disease epidemiology is more homogenous as compared to general animal population.

In the last few years, studies looking at the epidemiology of *C. burnetii* in camels in Kenya have shown high exposure levels to the pathogen in northern Kenya and provided evidence camels play a significant role in the epidemiology and transmission of *C. burnetii* to humans and other domestic animals [31, 40, 41]. Consequently, in the design of surveillance, prevention and control measures for this pathogen should take into account the growing camel population in this setting.

Significant differences in *C. burnetii* antibody seropositivity were observed in animals reared in different geographical locations (wards). Animals from Laisamis and Loiyangalani wards had 1.4 and 1.7 folds higher likelihood of being *C. burnetii* seropositive respectively compared to animals from Kargi/SouthHorr ward. Since animals from the region are all reared in a same system of nomadic pastoralism, other factors could have contributed to the observed heterogeneity in *C. burnetii* exposure levels. Although our study did not collect and incorporate environmental covariates as putative risk factors for *C. burnetii* seropositivity in animals, such environmental factors such as vegetation density, precipitation, wind speed and soil characteristics could play a role in explaining the observed differences in *C. burnetii* seroprevalence in animals across the different wards [6, 54]. Previous studies have explored the role of environmental factors in *C. burnetii* dispersal as documented during the outbreak in Netherlands [6, 55, 56], however spatial epidemiological studies on the role of environmental factors in *C. burnetii* dispersal are rare in the region, which limits our understanding of the role of environmental factors in Q-fever transmission dynamics in this setting.

The study was conducted in an area with high rates of undernutrition [57]. The relationship between infectious diseases and malnutrition has been shown to be bidirectional in which infections weaken the body's ability to fight diseases and cause malnutrition [58, 59]. However, data on the effect of zoonoses such as Q-fever on human nutrition outcomes is extremely rare in this setting [60]. Our study findings found no association between Q-fever seropositivity in humans and malnutrition. However the study only looked at exposure to *C. burnetii* and could not distinguish past exposure and active infection of Q-fever, hence cannot rule out the influence of Q-fever infection on human nutritional status.

Our study has several limitations. We used an indirect IgG ELISA to test the presence of antibodies against *C. burnetti* and could not distinguish between historical exposure and active infections. Additionally the tests used had less than 100% sensitivity and specificity which could pose a risk of misclassification. Our study population was mainly female animals providing milk to the households and children under five years and women of reproduction age. Although this population could provide valuable information on disease transmission and exposure levels for this population segment, the estimates may not be representative of the general population as not all ages and gender are included in the study. Our study did not include environmental factors as covariates when investigating factors associated with Q-fever antibody seropositivity, which may have accounted for some of the observed variations across different geographical study regions. Our study sampled only female and children for the humans which does not provide a complete randomized profile of human populations in the survey area. Although this was informed by the need to link the disease burden data with maternal and child nutritional data, future works should aim at sampling all age groups and gender in this setting.

A key strength of our study is the use of One Health concept by simultaneously assessing Q-fever in people and their livestock. In this case, we do not find evidence of household level association between levels of exposure to *C. burnetii* in livestock and people. However, this finding is biologically plausible given that the main mode of transmission for *C. burnetii* is inhalation of aerosals from conterminated environment, hence human exposure could occur even outside the household level given the disease endemicity in the region.

Increased risk of adverse pregnancy outcomes such as abortions and other reproductive disorders have been reported among women infected with *Coxiella burnetii* in previous studies [61]. In this setting were high levels of *C. burnetii* exposure in women was reported, further investigation on possible effect on *C. burnetii* infection on reproduction in women should be explored.

## 5 Conclusions

This study reported the exposure to Q-fever in humans and livestock among the pastoral community in Marsabit, Northern Kenya. Our results indicated that Q-fever is endemic in this setting, although the disease is neglected and not part of the diseases considered in surveillance and routine diagnosis at health facilities and veterinary diagnostic laboratories. Further studies designed in a One Health approach and utilizing molecular diagnostic tests to identify active *C. burnetii* infection are required to identify factors modulating *C. burnetii* burden and transmission dynamics and its effects on health and nutrition in humans in this setting. Such evidence will be beneficial in setting the country's disease control and prevention strategies.

## Declarations

### Author contribution statement

Josphat Muema, SM Thumbi, Mutono Nyamai: conceived and designed the experiments; performed the experiments; analyzed and interpreted the data; wrote the paper.

Nick Wheelhouse, Joseph Njuguna, Christine Jost: conceived and designed the experiments; contributed reagents, materials, analysis tools or data; wrote the paper.

Julius Oyugi: conceived and designed the experiments; performed the experiments; wrote the paper.

Zipporah Bukania conceived and designed the experiments; wrote the paper.

Brian Ogoti, Harriet Oboge performed the experiments; wrote the paper.

Anita Makori analyzed and interpreted the data; wrote the paper.

Maria del Pilar Fernandez analyzed and interpreted the data; contributed reagents, materials, analysis tools or data; wrote the paper.

Sylvia Omulo, conceived and designed the experiments; analyzed and interpreted the data; wrote the paper.

### Funding statement

This work was supported by Office of Technical and Program Quality, Bureau for Humanitarian Assistance, U.S. Agency for International Development [720FDA18IO00035], Fogarty International Center and the Institute of Allergy and Infectious Diseases of the National Institutes of Health [D43TW011519].

### Data availability statement

Data will be made available on request.

### Declaration of interest’s statement

The authors declare no conflict of interest.

### Additional information

No additional information is available for this paper.
