# Supplementary material for: Endemicity of Coxiella burnetii infection among people and their livestock in pastoral communities in northern Kenya
Source: Heliyon. 2022 Oct 21;8(10):e11133. doi: 10.1016/j.heliyon.2022.e11133 (PMC9593183; doi:10.1016/j.heliyon.2022.e11133)
Supplement: Supplementary file [file mmc1.docx]

Annex 1: human sample collection and tracking questionnaire

HUMAN SAMPLE TRACKING SHEET

Location: __________________________ Household ID: ________________Date: ______________

Study Participant (Mother, Child): **____________________** Staff: **_______________________**

| **#** | | **Sample ID**  **(Barcode)** | | **Study participant** | | **Sample type** | | **Study visit Type** | | **Sex** | **Age** | **Physiological status** | | | |
| --- | --- | --- | --- | --- | --- | --- | --- | --- | --- | --- | --- | --- | --- | --- | --- |
| 1 | | Place barcode here | |  | |  | |  | |  |  |  | | | |
| 2 | | Place barcode here | |  | |  | |  | |  |  |  | | | |
| 3 | | Place barcode here | |  | |  | |  | |  |  |  | | | |
| **Sample type**  Serum - SR  Whole blood - WB  Blood Clot - BC | | **Study visit type**  1 – Baseline  2 – Six months’ visit  3 – Twelve months’ visit | | **Sex**  Male - M  Female - F | |  | | **Physiological status:**  P - Pregnant  L – Lactating  NP/NL – Not pregnant & Not Lactating  PL – Pregnant & Lactating | | | |  |  |  | |

Annex 2: Animal level sample collection and tracking questionnaire

ANIMAL SAMPLE TRACKING SHEET

Location: _________________ Household ID: ________ Date: _______

Species (Bovine, Goat, Sheep, Camel): ____________ Staff: _____________

| **#** | | Sample ID  (Barcode) | | Species | Sample type | Study visit Type | | Breed | Sex | | Age | Physiological status | History |
| --- | --- | --- | --- | --- | --- | --- | --- | --- | --- | --- | --- | --- | --- |
| 1 | | Place barcode here | |  |  |  | |  |  | |  |  |  |
| 2 | | Place barcode here | |  |  |  | |  |  | |  |  |  |
| 3 | | Place barcode here | |  |  |  | |  |  | |  |  |  |
| **Sample type**  Serum - SR  Whole blood - WB  Blood Clot - BC  Milk - MK | | **Study visit type**  1 – Baseline  2 – Six months’ visit  3 – Twelve months’ visit | | **Sex**  Male - M  Female - F | | **Breed**  A - indigenous  B – Exotic  C - Crossbreed | | |  | | | |  |

**Age(cattle):** A= 2-3 yrs, B= 3-4yrs, C = 4-5yrs D = >5yrs

**(sheep/goats):** A= <1yr, B=1-2yrss,

C= 2-3yrs D=3-4yrs E=>4yrs

**(Camels):** A= <4yrs B =4-6yrs C= >6yrs

**History:** 1 = Abortions 2 = still births 3 = weak young

4 = Metritis

5 = Retained placenta

6 = Swollen joints
